# Interactive *K*-Means Clustering Method Based on User Behavior for Different Analysis Target in Medicine

**DOI:** 10.1155/2017/4915828

**Published:** 2017-10-26

**Authors:** Yang Lei, Dai Yu, Zhang Bin, Yang Yang

**Affiliations:** ^1^College of Computer Science and Technology, Northeastern University, Shenyang, China; ^2^College of Software, Northeastern University, Shenyang, China

## Abstract

Clustering algorithm as a basis of data analysis is widely used in analysis systems. However, as for the high dimensions of the data, the clustering algorithm may overlook the business relation between these dimensions especially in the medical fields. As a result, usually the clustering result may not meet the business goals of the users. Then, in the clustering process, if it can combine the knowledge of the users, that is, the doctor's knowledge or the analysis intent, the clustering result can be more satisfied. In this paper, we propose an interactive *K*-means clustering method to improve the user's satisfactions towards the result. The core of this method is to get the user's feedback of the clustering result, to optimize the clustering result. Then, a particle swarm optimization algorithm is used in the method to optimize the parameters, especially the weight settings in the clustering algorithm to make it reflect the user's business preference as possible. After that, based on the parameter optimization and adjustment, the clustering result can be closer to the user's requirement. Finally, we take an example in the breast cancer, to testify our method. The experiments show the better performance of our algorithm.

## 1. Introduction

Custer analysis is an unsupervised learning method which is used to explore the interrelationships among a collection of patterns by organizing them into homogeneous clusters. However, when putting this kind of analysis technique into the big data field, sometime it may be difficult to find a satisfied result not only as a result of the high feature dimensions, but also as a result of the different analysis target. It is the fact that different users may have different analysis targets of business objectives [1]. Then, when we evaluate the result of the clustering algorithm, we also want to know whether the result can reflect the user's intent or not [2].

Normally, a clustering algorithm always needs the following steps to deal with the data: load a data set, select a few parameters, run algorithm, and then view the consequence. In this process, clustering is used simply to analyze the data rather than explore it. Then, when the clustering process wants to meet the analysis target, it needs the users to predefine the input parameters. Then, when the parameter is not set properly, the results may not be satisfied. That is to say, the business semantics of clustering results does not meet the analytical target. For example, for medical disease data, the medical researchers may be more concerned about the symptoms related to a disease, and the medical workers in the face of the same data may be of more sense of disease and the disease treatment, and others may be more interested in the properties of drug effects. That is to say, analysis target of different user on the same data may be different. One of the reasons for this is that, under the environment of big data, facing high-dimensional and complex data, the degree of concern and attention of different users for the data attributes of same data may be different. Therefore, as to different users, the importance of the same attributes may be different, as for the reason of different analysis target. Then, sometimes the clustering result which does not consider the business goal of the users may not be satisfied. In this case, someone may argue that the attributes which are not very important can be filtered out before the clustering. However, sometimes the users want to observe multiple attributes which may be interleaved together and many of the users do not know exactly how to filter out the attributes when the number of the attributes is in a large body. Then, filtering out the attributes manually done by the normal users before the clustering process is always very difficult. In this situation, modifying the input parameters directly and then rerunning the algorithm [3] always become a way to solve this problem. However, this method is always time-consuming, inconvenient, and nonintuitive. What is more, there are rarely recommendations about settings of these parameters. That is to say, for a normal people, modifying the parameters manually can be a difficult task since they may not know which parameters should be modified and to what extent the parameters can be modified to meet the analysis target. Then, if there is a recommended parameter setting, it will be a kind of references which can be helpful for users.

Therefore, we propose an interactive method which is based on the analysis of the interactive behavior of the users on the clustering result. By doing so, the analysis intent of the users can be captured and the parameters related to the *K*-means clustering algorithm will be adjusted accordingly. In summary, the contribution of this paper includes the following.

(1) We present an interactive framework especially for the *K*-means clustering algorithm. The core of this framework is the interaction with the users in order to get their analysis intent and adjust the parameters automatically.

(2) The interaction between the users and the clustering result can reveal the intent of their analysis goal. Then, the interactive behavior model which describes the user's operation on the clustering result is defined. Then, based on this model, one typical kind of the user's behavior is analyzed.

(3) We propose a method for getting the recommended parameters to be adjusted in the *K*-means algorithm.

## 2. Related Works

Clustering in data mining falls into the category of unsupervised learning, which is to find the interrelationship between the data. As such, the primary purpose in data mining is to gain insights into the distribution of the data and to understand the characteristics of each cluster. Clustering in data mining can be achieved in several ways. Literature [4–7] suggests distinguishing among the different types of methods.

Partitioning methods [8] aim to organize data into a number of distinct clusters, such that the total deviation of the data points from the cluster centers is minimized. In general, methods based on partitioning are sensitive to noise and clusters are regularly shaped and requiring the number of resulting clusters as an input parameter, even though this type of algorithm is scalable to the number of objects. Probably the most popular method of this type is the *K*-means algorithm for clustering, which requires the number of clusters (*K*) as an input parameter [9]. And Hierarchical methods [10] are based on a recursive split of the data into a tree structure. In general, methods of this type are scalable with the number of objects and the resulting clusters can have arbitrary shapes. Density-based clustering methods [11] regard clusters as regions with high densities of objects, separated by sparse areas. Such methods can be used to efficiently filter noise and can discover arbitrary-shaped clusters. These clusters may have an arbitrary shape and the points inside a region may be arbitrarily distributed, but knowing the distribution and density might be a prerequisite to achieve meaningful clustering results. Besides, there is also another type named grid-based.

For improving the satisfaction of the clustering results, there are some kinds of methods to adjust the clustering results for the above clustering algorithm. Some methods adjust the clustering result by modifying the parameters of the clustering algorithm, such as the number of target clusters and the density or distance threshold for partitioning the clusters as well as the weights of each attribute when computing the similarity between each data point. However, most the current methods modify the parameters directly by the users themselves. Sometimes users do not know well about the algorithm, and they just use it in analysis. Then, it is impossible to correctly modify the parameters to meet the business goal. Besides, some works depend on the expert to initially set the parameters. But as in a big data field, the number of the vectors of the data will be large. Different user may focus on different aspect of the data as they may be from different domain. Then, listing all the potential parameters in every domain is hard and not flexible. For solving this problem, it needs to consider the user's target in the clustering process.

Then, in this paper we propose an interactive *K*-means clustering algorithm, the core of which is to adjust the parameter in the algorithm according to the user behavior. Then, by the analysis of the user behavior, the intent of the analysis business of the user can be predicted and the parameter recommendation according to such intent can be done. Then, the clustering result can be much more satisfied by the users.

## 3. Method Overview

**Figure 1:**
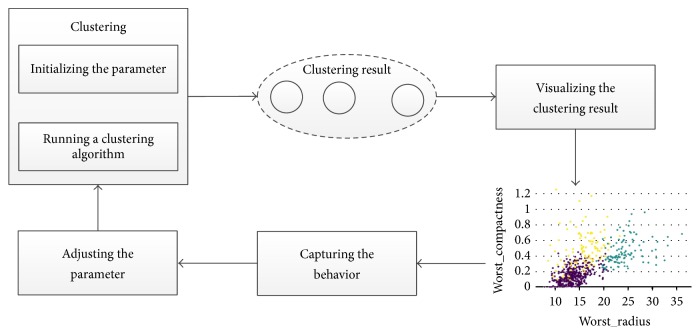
Framework of the interactive clustering method.

As for different users who may have different analysis requirements of business objectives, in order to meet all kinds of analysis requirements of users, this paper presents a method for adjusting clustering results according to the user's behavior. The basic framework of the method is shown in Figure 1. In this framework, there are 4 main components:* clustering*,* visualizing the clustering result*,* capturing the behavior*, and* adjusting the parameters*.* Clustering *is used to run the *K*-means algorithm with the needed parameters fed into the algorithm. At the beginning, the parameters are set by the default value or by an expert. Then, by the component* visualizing the clustering result*, the clustering result will be visualized. The users can view the result and adjust the place of some data point or the weight of some attribute. All of this behavior will be recorded by the component* capturing the behavior*. Then, the component* adjusting the parameters *can analyze the behavior on a small part of the data points to compute the recommended parameters to adjust the clustering result by feeding the parameters to the clustering algorithm again until the result will be satisfied by the users.

Then, in the following, we will discuss the implementation of the key components. As for the visualization part we can use R or d3.js to help us; then we will not discuss it too much. As for the component* clustering*, in this paper, we does not modify the internal mechanism of *K*-means algorithm, and then we will only discuss the way of how to get the initial setting of the parameters used in this algorithm. In this paper, we discuss the way to set the weights of each attribute. Since we want to optimize the settings of the parameters, we will present the way for analyzing the user's behavior to generate the recommended ones.

### 3.1. Initializing the Weights in *K*-Means Algorithm

The importance of the attributes may be different in an analysis task. Then, in this paper, we present a method for computing the weights in the *K*-means algorithm.

Given *X* = {*x*_1_, *x*_2_,…, *x*_*n*_} is a data set with *n* samples, in which *x*_*i*_ = [*x*_*i*1_, *x*_*i*2_,…, *x*_*im*_] represents a data object (sample) with *m* classification attributes and *x*_*ip*_ represents the value of the attribute *P* in the object (sample) *x*_*i*_. We apply the *K*-means algorithm based on attribute weighting to divide data into *K* clusters.

Assuming that the weight of each attribute is *w*_1_, *w*_2_,…, *w*_*m*_, and *w* ≥ 0, *j* = 1,2,…, *m*. The weighted data objects are as follows: *x*_*i*_′ = *x*_*i*_*∗w*, *i* = 1,2,…, *n*. The initial weight of clustering process is applied to the method of variance coefficient which is commonly used in statistics. The method can determine a weight value *w*_*k*_ of each attribute by using the method of coefficient of variation and empower. The specific steps are as follows:

(1) Calculating the standard deviation of the attribute *P* in samples:

(1) $$\begin{array}{c} \sigma_{P}=\sqrt{\frac{\sum_{i=1}^{n}\left(x_{iP}-\bar{x_{P}}\right)}{n-1}},\;\left(P=1,2,\ldots ,m\right), \end{array}$$

where $\bar{x_{P}}$ is the average value of the *P* attribute of the sample, $\bar{x_{P}}=\sum_{i=1}^{n}x_{iP}/n,\;\;(P=1,2,\ldots ,m)$.

(2) Calculating the coefficient of variation of each attribute:

(2) $$\begin{array}{c} {CV}_{P}=\frac{\sigma_{P}}{\bar{x_{P}}},\;\left(P=1,2,\ldots ,m\right). \end{array}$$

The coefficient of variation reflects the relative variation degree of each attribute, and the greater the coefficient of variation, the greater the change of the attribute in the sample.

(3) Normalizing the coefficient of variation of each attribute, and then the weight of each attribute *w*_*P*_ is determined.

(3) $$\begin{array}{c} w_{P}=\frac{{CV}_{P}}{\sum_{i=1}^{m}{CV}_{i}},\;P=\left(1,2,\ldots ,m\right). \end{array}$$

The weight of *w*_*P*_ reflects the extent of the different attributes to which it can affect the clustering result. The above method can only find the weight according to the distribution of each attribute. Then, for the different analysis task, different attribute may receive different attentions. In the following, we show how to modify the clustering result according to the user's satisfaction.

### 3.2. Interactive Behavior on the Clustering Result

During the clustering process, there may be deviation between the clustering results, the fact or the user's business filed using initial weight. Probably users cannot get satisfied clustering results at a time. For example, a data *x* is divided into cluster “A” using clustering algorithm, but in accordance with user's knowledge it should be divided into “B.” In order to reduce the deviation, we construct a model for user to adjust the clustering result. An interactive behavior can be described with the following vector: IB = 〈*Q*, *q*_0_, M, MH, SH, *F*〉, where

- *Q* is finite state set, which can be defined as *Q* = {S_START, S_END, S_DRAG, S_CANCEL};
- *q*_0_ is initial state, in *Q*; *q*_0_ = {S_START};
- M is the set of received messages. It only can receive one message at a time which can be defined as M = {WM_LBUTTONUP, WM_LBUTTONDOWN, WM_ONMOUSEMOVE};
- MH is the set of finite message handling. It corresponds to the message set one by one; the main completion is state transition. It is equivalent to the state transition function, which can be defined as MH = {OnLButtonDown(), OnLButtonUp(), OnMouseMove()};
- SH is the set of state handling function. It is responsible for the functional operation of state handling, and it is a set of actions when a state transition occurs, which can be defined as SH = {DoGetFirstPt(), DoGetFirstCt(), DoGetEndCt(), DoEnd(), DoMove(), DoCancel()};
- *F* is termination state; it is also contained in *Q*, *F* = {S_END, S_CANCEL}.

In this paper, we focus on an interactive behavior, and in the following, we will introduce it.* Interactive Behavior with Changing the Cluster Directly* can be defined as the 6-vector model: PSOIB = 〈*Q*, *q*_0_, M, MH, SH, *F*〉. In this model, the function DoMove() as mentioned above means that users can move a point from one cluster to another one. Then, by this behavior, users can choose the data points which are divided into the wrong cluster in the user view and move them to the right cluster. For example, there are *n* data points which are regarded as locating in the wrong groups (*scl*_1_, *scl*_2_,…, *scl*_*n*_) according to the user's analysis target. Then triggering event DoMove(), these data points can be moved to the other cluster which they should belong to.

**Figure 2:**
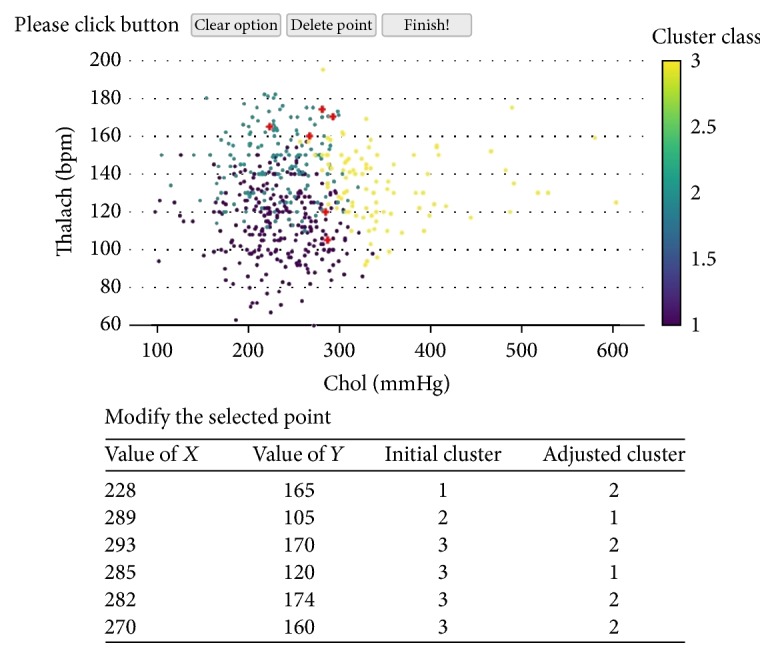
Example of interactive clustering.

More specifically, users can move mouse to look at the detail of each data point in order to get a preliminary understanding. Then they may find out some wrongly assigned points in some cluster according to their domain knowledge or different analysis target. Then, the users can select the points by pressing the left mouse button which can trigger the event OnLButtonDown(). Then, the event can help to record the initial state of the belonging relations between the data points and the clusters. Then, the users can move the selected data points to the other cluster which can trigger the events OnMouseMove() and DoMove(). If the users want to put the data point into some cluster, they can move the data points to the cluster and release the left mouse button which will trigger the event OnLButtonUp(). Then, when the event OnLButtonUp() is triggered, the new weights of the attributes will be automatically computed according to the following PSOIB algorithm. For example, as shown in Figure 2, there are 6 selected points with red color which are (228, 165), (289, 105), (293, 170), (285, 120), (282, 174), and (270, 160) and their initial clusters are 1, 2, 3, 3, 3, and 3, respectively. According to analysis aim, the user relocates these points to clusters 2, 1, 2, 1, 2, and 2 by moving mouse. Then, the PSOIB algorithm will compute the new weights according to the users' adjustment.

We just list one typical interaction behavior in this paper. In fact, one can define other interaction behaviors by refining the interactive behavior. After collecting the user's behavior, we need to analyze the behavior in order to get the user's analyzing intent. Since the big data may have a large body of attributes, in an analysis activity, different users may have different business goal. For example, as for analyzing the diabetes, some nutritionists aim at finding the relation with the diabetes and the eating habits of a person, while other nutritionists focus on the relation with the diabetes and the genes of a person, which may result in the different weights fed into the *K*-means algorithm. Then, in the following, we will discuss how to adjust the weights in order to meet the requirement of the users according to their interactive behavior.

### 3.3. Adjusting Weights with PSOIB

In the PSOIB* (Interactive Behavior with Changing the Cluster Directly)*, according to different analysis goals, users can choose the point which is divided into the wrong cluster and move it to the cluster which is thought to be the right cluster by the user. Then, by this kind of operations, the importance of the attributes can be revealed since the similarity degree between one point and another always relates to the weights and the result of such operation reveals that the similarity degree will be different as a result of the change of the importance. Then, the weights for each attribute must be recomputed to optimize the clustering result. In the following, we will present the method to recompute the weights according to this behavior.

Then, imagine that the number of the attributes of a data point is *k* and the attributes form a set *A* = {*a*_1_, *a*_2_,…, *a*_*k*_}, where *a*_*i*_ is an attribute. The initial weights of the corresponding attributes can form a set *W* = {*w*_1_, *w*_2_,…, *w*_*k*_}, where *w*_*i*_ is the weight of the attribute *a*_*i*_ in the set *A*. The distance between the data points is always computed by a weighted Euclidean distance formula which can be used in a clustering algorithm to evaluate the similarity between different data points. Then, the weights of the attributes always play an important role in getting the better clustering result. Initially, the weights may neglect the business goal of the users which may result in a dissatisfied clustering result. Then, the weights will be adjusted to meet the analysis target. When the users select a data point and move it from one cluster to another, it means that the data point is misclassified according to user's intention. For a data point *x* which is misclassified, imagine that the original cluster it belongs to is *scl*_*x*_ with the cluster center as *sc*_*x*_. Imagine that the new cluster it belongs to is *scl*_*x*_′ with the cluster center as *sc*_*x*_′. The movement of the point* x* from the cluster *scl*_*x*_ to *scl*_*x*_′ means that the distance between *x* and *scl*_*x*_ should be larger than the distance between *x* and *scl*_*x*_′. Then, the weights should be adjusted to satisfy this constraint. The problem for adjusting the weights can be described as follows.

Imagine that there are *n* data points which form a set *D* = {*d*_1_, *d*_2_, …, *d*_*n*_}. The weights to be recommended form a set *W*′ = {*w*_1_′, *w*_2_′, …, *w*_*k*_′}. Then, the problem of getting the weights *W*′ can be shown in formula

(4) Min ∑idisdi,di·scl′,W′disdi,di·scl,W′,

where *d*_*i*_ · *scl* is the cluster center of the data point *d*_*i*_ before the movement. *d*_*i*_ · *scl*′ is the cluster center of the data point di after the movement. If the data point *d*_*i*_ has not been moved, then *d*_*i*_ · *scl* and *d*_*i*_ · *scl*′ are the same.

The above problem is to find a combination of the weights of the attributes to make formula (4) the minimum which can be seen as a continuous space optimization problem. In this paper, we use PSO algorithm to solve this problem.

We define the swarm particles as points inside the weight space, that is, *n*-dimensional vectors in the weight space. Given a number *m* of particles, we initialize the swarm by associating each particle to one of the *m* nearest numbers of the original weights *W*. Then, we generate a random speed vector Δ*p*_*k*_ independently of each swarm particle *p*_*k*_ to initialize the stochastic exploration.

One of the most important points in an optimization process is the definition of the fitness function, which should represent the effectiveness of the solution reached by the swarm particles. Taking into our optimization problem, formula (5) defines the fitness function that expresses the fitness associated with the solution found by the generic particle *p*.

(5) $$\begin{array}{c} fitness\left(\;p\;\right)=\sum_{i}\frac{dis\left(d_{i},d_{i}\cdot scl^{\prime },p\right)}{dis\left(d_{i},d_{i}\cdot scl,p\right)}. \end{array}$$

Then, the PSO algorithm can find the optimal combination of the weights of each attribute.  Algorithm 1 presents the PSO algorithm to find the optimal combination of the weights which can meet the user's satisfaction.

Based on the above algorithm, we can find the optimal combination of the weights. However, the change of such adjusting on the weights may cause the fact that the number of the clusters should also be changed. Then, before reclustering by using the adjusting weights, we also need to adjust the number of the clusters since after including some data points, the cluster may contain much more data points, which may be seemed different from others. Then, a decision should be made to suggest whether to change the number of the clustering or not. In this paper, we use degree of cohesion (DCI) and degree of separation between clusters (DSB) to evaluate how to change the number of the clusters.

Suppose that there is a *p*-dimensional data set *D* = {*x*_1_, *x*_2_, …, *x*_*n*_}, where *n* is the number of data objects, and *X* = {*X*1, *X*2,…, *Xp*} represents a data point. Then data set *D* was divided into *i* subsets which is described as *D* = {*C*_1_, *C*_2_,…, *C*_*i*_}  (*i* = 1,2,…, *k*). *C*_*i*_ is the cluster, of which *t*_*i*_ represents the center. And *n*_*i*_ records the number of data points in cluster *C*_*i*_.

The degree of cohesion in the cluster (DCI) means the degree of discretization of the data set. The smaller the value of DCI, the higher the similarity in the cluster and then the better the clustering results. The DCI value, which is described as formula (6), is measured by calculating the degree of deviation from each point in the cluster to the center of the cluster.

(6) $$\begin{array}{c} DCI=\underset{x\in C_{i}}{\sum }{d\left(t_{i},x\right)}^{2}=\frac{1}{2n_{i}}\underset{x\in C_{i}}{\sum }\;\underset{y\in C_{i}}{\sum }{d\left(x,y\right)}^{2}, \end{array}$$

where *d*(*x*, *y*) means the distance between objects *x* and *y*. It can be calculated as formula

(7) $$\begin{array}{c} {d\left(x,y\right)}^{2}=\overset{p}{\underset{i=1}{\sum }}w_{i}{\left\|\;x^{i}-y^{i}\;\right\|}^{2}. \end{array}$$

If the value of DCI is lower than the one before adjusting the weights, the adjusted weight based on user's intention not only enhances the importance of analysis goals but also ensures the reliability of cluster results. On the other side, if the value of DCI outweighs the preadjusted one, it indicates that the condensation degree within clusters declined. When the DCI value exceeded a certain extent, it indicates that the current cluster may contain two or more clusters of the user's target clustering results. In this case, the number of the clusters should be added one.

The other measure to value the clustering results is the degree of separation between clusters (DSB) defined as formula (8), which means the difference between the two clusters. Furthermore, the greater the value of the DSB, the larger the difference between the two clusters. Therefore, the smaller the similarity, the better the clustering results.

(8) DSBCi,Cj=121ni∑x∈Cidx,tj2+1nj∑x∈Cjdx,ti2.

The value of DSB is measured by calculating the mean value between the degree of deviation from each point in the cluster *C*_*i*_ to the center of the cluster *C*_*j*_ and the degree of deviation from each point in the cluster *C*_*j*_ to the center of the cluster *C*_*i*_. The distance calculation formula is the same as formula (7). The value of DSB(*C*_*i*_, *C*_*j*_) is equal to the value of DSB(*C*_*j*_, *C*_*i*_). By contrast with DCI value, the larger the DSB value, the greater the difference between clusters. In this case, the smaller the similarity between clusters, the better the clustering results, which means that the adjusted weight can be applied to carry out a better result with the purpose of the user. Provided the DSB value decreased, it shows that the current result may not reliable. When the value of DSB is smaller than the predefined threshold, the two clusters *C*_*i*_ and *C*_*j*_ should be combined together since they are very similar based on the analysis goal. Therefore, we recommend combining these clusters. In this case, the number of the clusters should be minus one.

## 4. Evaluation

**Figure 3:**
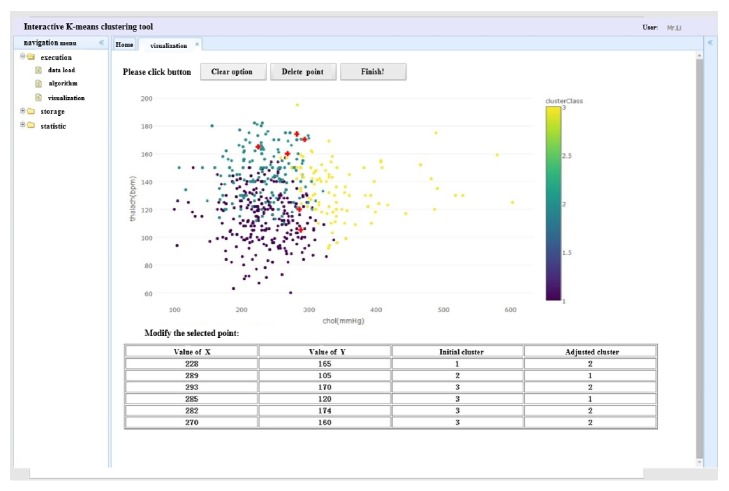
The prototype system of interactive processing.

Based on the process of our method, we design a prototype seen in Figure 3. In this prototype, in order to optimize the clustering results, the user's analysis target and business requirement are taken into account to assist user adjusting the weights in the clustering process. In this process, firstly, the data to be analyzed will be uploaded. Then, the system firstly uses the *K*-means algorithm by setting the input parameters according to the previous experience. Then, the result will be visualized in the system. The users can explore the data points according to their business interest. If they want to change the clustering result to meet their analysis target, they can adjust the clustering results interactively. For example, if they want to move the date points (228, 165), (289, 105), (293, 170), (285, 120), (282, 174), and (270, 160) from their initial clusters with number of 1, 2, 3, 3, 3, and 3, respectively, to clusters with number of 2, 1, 2, 1, 2, and 2, they will select these data points by pressing the left mouse button and move these data points to the corresponding clusters by releasing this button. Then, the PSOIB algorithm will compute the new weights according to the users' adjustment. Before reclustering by using the adjusting weights, the number of the clusters may also be adjusted. The degree of cohesion (DCI) and degree of separation between clusters (DSB) will be computed to evaluate how to change the number of the clusters. Then, the new weights and the number of the clusters will be fed into the clustering algorithm as the input to recluster according to the user's analysis target. Then, the result will be visualized again and if the users are not satisfied with the result, the interactive clustering process will be done again.

To verify the effectiveness of our interactive clustering method, we do experiments with breast cancer data set in UCI [12] which includes 569 data and 32 attributes. The first and the second dimensional attributes are the patient number and the diagnostic information. And from the third dimensional attribute to the thirty-second attribute, respectively, it is the average, standard, worst, or maximum of the radius, texture, perimeter, area, smoothness, compactness, concavity, pits, symmetry, and fractal dimension. The user performs an interactive *K*-means clustering analysis on breast cancer data.

**Figure 4:**
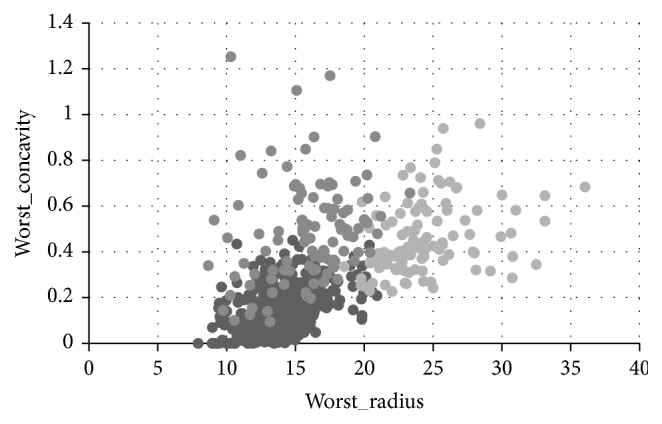
Clustering results with analysis purpose of worst_radius and worst_concavity.

**Figure 5:**
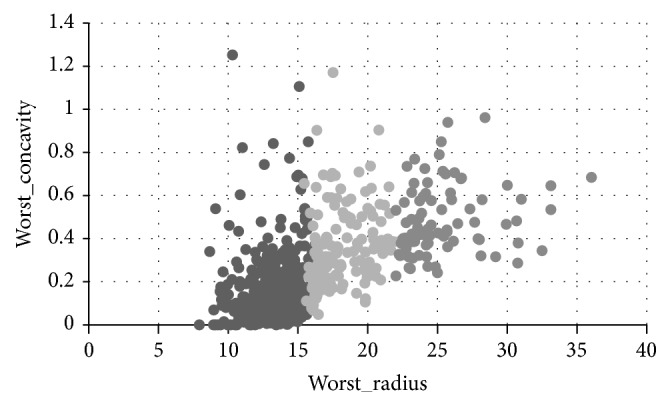
Interactively clustering results with clustering number of 3.

The purpose of analyzing is concentrated on the effect of worst concavity and worst radius which are the attributes of the data set.  Figure 4 shows the clustering result by *K*-means clustering analysis of the breast cancer data when the number of clusters is set to 3.  Figure 5 is the clustering result after the adjustment of the weights according to the user's behavior. Based on user's analysis goals, the user selects 6 points in Figure 4 which are seen as locating in the wrong cluster. They are (13.15, 0.0942), (11.69, 0.01256), (23.32, 0.6566), (18.55, 0.3355), (9.773, 0.1434), and (20.38, 0.5036), which belong to cluster (2, 2, 2, 3, 2, 1), respectively. Then those points are adjusted to the cluster (1, 1, 3, 2, 1, 2). This interactive operation indicates that those points are more similar to the points in the adjusted cluster than the points in previous cluster. Following step would be adjusting weight by PSO algorithm to get a set of adjusted weights which are changing from (0.033, 0.065) to (0.1, 0.16).

From the comparison of Figures 4 and 5, it is observed that the clustering result by interactive adjusting is more satisfied based on user's analysis goals, since the data points in each cluster are more concentrated, the density is higher, and the circle is smaller. This result shows that the overall quality of the clustering results is improved, and the clustering results are optimized by the user's interaction.

**Figure 6:**
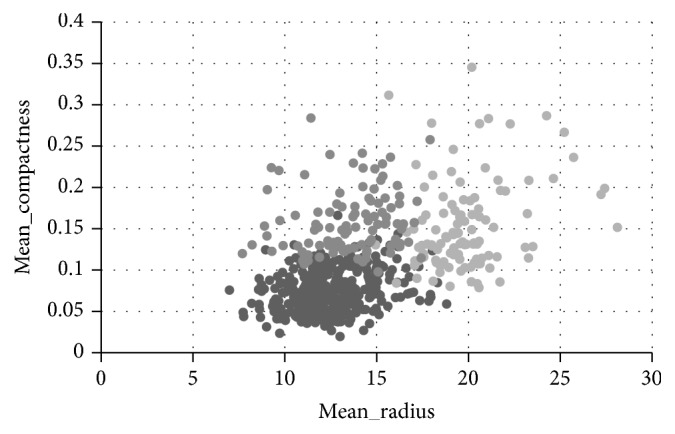
Clustering results with analysis purpose of mean_radius and mean_compactness.

**Figure 7:**
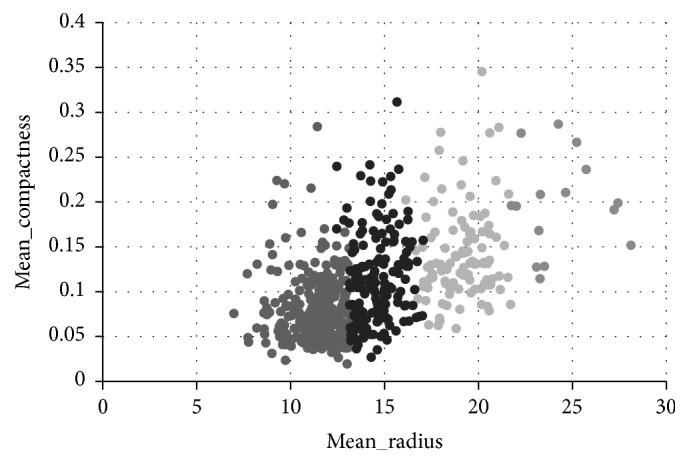
Interactively clustering results with clustering number of 4.

Then the purpose of analyzing is replaced by the effect of mean compactness and mean radius.  Figure 6 shows the clustering result by *K*-means clustering analysis of the breast cancer data when the number of clusters is set to 3.  Figure 7 is the clustering result after the adjustment of the weights. According to user's analysis aim, five points are chosen as the wrong points which are not so similar to the others in current cluster when we look at Figure 6. The selected points are (12.68, 0.1262), (19.1, 0.1791), (17.02, 0.1496), (17.06, 0.1056), and (13.14, 0.1089), which belong to cluster (3, 1, 1, 1, 3), respectively. Then those points are adjusted to the cluster (1, 2, 2, 2, 1). The following step, similar to last experiment, would be adjusting weight by PSO algorithm to get a set of adjusted weights. The weights of two goals are changing from (0.34, 0.32) to (0.32, 0.37). Before reclustering, the interactive behavior has been analyzed by calculating the value of DCI and DSB. The DSB value between two in three clusters is larger than the threshold with all the values increasing, while the DCI value of cluster 2 is larger than before and even over the threshold, which means that the current cluster may contain two or more clusters of the user's target clustering results. Therefore, split recommended that this cluster should be divided into two parts, and the user is satisfied with the split result; at the end of process, reclustering is executed with both clustering number of 4 and the adjusted weights.

From the comparison of Figures 6 and 7, it is observed that the clustering result by interactive adjusting is more satisfied, because the data points with the same group are closer in Figure 7, which means that the clustering result can reflect the characteristics in the same group much more accurately. However, in the Figure 6, the data points in different group are always overlapped together, which means that the clustering cannot reflect the characteristics of the group.

## 5. Conclusion and Discussion

In this paper, we proposed a novel approach to apply interactive behavior to clustering process. The main feature of our method is to analyze the user's behavior to adjust the parameters in a clustering algorithm in order to get the meaningful result. Then, users can adjust the parameter, for example, weight, in a clustering algorithm, according to their knowledge in the business field. We evaluated our approach on a suitable data set with known best clustering results depending on field knowledge. In this way, we compared the traditional clustering results and adjusted clustering results to verify the effectiveness of our method. Finally, we demonstrated the usefulness of our interactive approach that results are very close to the results of user expectations in practical application.

Nevertheless, we must mention the shortcomings and weaknesses we discovered in our approach. The evaluation criteria to clustering results of this method are the user's domain knowledge. Only by fully understanding the clustering results, the domain experts can better apply domain knowledge for the analysis of a given domain to make right decision. Although we provide graphics visual interface to display the results of clustering for increasing the interpret-ability, it is still difficult for users to understand the meaning of clustering results intuitively, because the visualization of the clustering result only presents the clustering label of each data point but not gives enough business meaning of the cluster centers.

Out of this consideration, we must and will consider further approaches to address feature extraction and knowledge interpretation. For the low-dimensional data (lower than 3), it can be expressed by a graphical visualization methods, but for high-dimensional data, it is hard to be directly visualized. In general, people's understanding of high-dimensional graphics is also very difficult. People usually adopt the method of dimension reduction for visualization, but this method may cause data distortion, leading to the visualization results far away from the clustering results with real data. For the convenience of people's understanding of the clustering results, it is necessary to extract knowledge from the result of clustering by feature attributes extraction and knowledge interpretation.

## Figures and Tables

**Algorithm 1:**
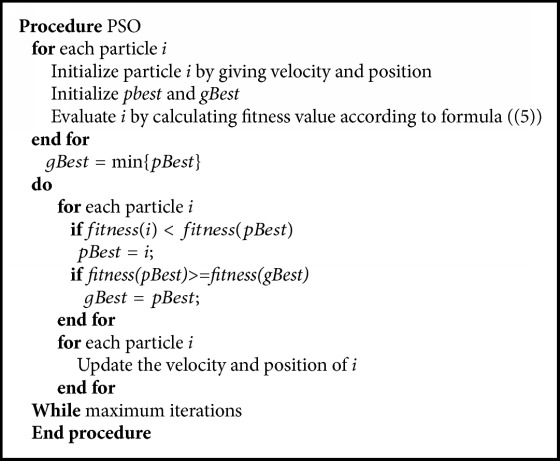
PSO algorithm to find the optimal combination of the weights.
